# A randomized trial on effectiveness of artemether-lumefantrine *versus *artesunate plus amodiaquine for unsupervised treatment of uncomplicated *Plasmodium falciparum *malaria in Ghanaian children

**DOI:** 10.1186/1475-2875-7-261

**Published:** 2008-12-19

**Authors:** Robin Kobbe, Philipp Klein, Samuel Adjei, Solomon Amemasor, William Nana Thompson, Hanna Heidemann, Maja V Nielsen, Julia Vohwinkel, Benedikt Hogan, Benno Kreuels, Martina Bührlen, Wibke Loag, Daniel Ansong, Jürgen May

**Affiliations:** 1 Infectious Disease Epidemiology, Bernhard Nocht Institute for Tropical Medicine, Hamburg, Germany; 2 Ministry of Health/Ghana Health Service, District Health Directorate, Agona, Afigya Sekyere District, Ghana; 3 Kumasi Centre for Collaborative Research in Tropical Medicine, Kumasi, Ghana; 4 Agogo Presbyterian Hospital, Agogo, Asante Akim North District, Ghana; 5 Department of Child Health, School of Medical Sciences, Kwame Nkrumah University of Science and Technology, Kumasi, Ghana; 6 Department of Paediatrics, University Medical Centre Eppendorf, Hamburg, Germany; 7 Section for Tropical Medicine, Bernhard Nocht Clinic, University Medical Centre Eppendorf, Hamburg, Germany

## Abstract

**Background:**

Numerous trials have demonstrated high efficacy and safety of artemisinin-based combination therapy (ACT) under supervised treatment. In contrast, effectiveness studies comparing different types of ACT applied unsupervised are scarce. The aim of this study was to compare effectiveness, tolerability and acceptance of artesunate plus amodiaquine (ASAQ) against that of artemether-lumefantrine (AL) in Ghanaian children with uncomplicated *Plasmodium falciparum *malaria.

**Methods:**

A randomized open-label trial was conducted at two district hospitals in the Ashanti region, Ghana, an area of intense malaria transmission. A total of 246 children under five years of age were randomly assigned to either ASAQ (Arsucam^®^) or AL (Coartem^®^). Study participants received their first weight-adjusted dose under supervision. After the parent/guardian was advised of times and mode of administration the respective three-day treatment course was completed unobserved at home. Follow-up visits were performed on days 3, 7, 14 and 28 to evaluate clinical and parasitological outcomes, adverse events, and haematological recovery. Length polymorphisms of variable regions of *msp1 *and *msp2 *were determined to differentiate recrudescences from reinfections. Acceptance levels of both treatment regimens were assessed by means of standardized interviews.

**Results:**

Adequate clinical and parasitological responses after AL and ASAQ treatment were similar (88.3% and 91.7%, respectively). Interestingly, more late clinical failures until day 28 occurred in AL-treated children than in those who received ASAQ (17.5% and 7.3%, respectively; Hazard Ratio 2.41, 95% CI 1.00–5.79, p < 0.05).

Haematological recovery and drug tolerability were not found to be significantly different in both study arms. The acceptance of treatment with ASAQ was higher than that with AL (rank-scores 10.6 and 10.3, respectively; p < 0.05).

**Conclusion:**

Unobserved AL and ASAQ treatment showed high adequate clinical and parasitological responses, though AL was inferior in preventing late clinical failures.

## Background

Artemisinin-based combination therapy (ACT) is advocated as the way forward in antimalarial treatment to overcome the global spread of *Plasmodium falciparum *drug resistance [1]. ACT rapidly diminishes parasite biomass leading to clinical and parasitological cure while at the same time gametocytocidal activity might be able to reduce overall malaria transmission [2]. Initially, efficacy and safety of ACT had been evaluated in Southeast Asia [3], and only recently, data became available from randomized clinical trials in sub-Saharan Africa, where the highest malaria burden occurs in children under five years of age [4]. Based on recommendations of the World Health Organization (WHO), several sub-Saharan countries introduced ACT as first-line treatment for uncomplicated malaria, although the comparatively high costs of a treatment dose are a burden to their health systems [5]. Currently, artemether-lumefantrine (AL) and artesunate plus amodiaquine (ASAQ) are the only widely available drugs, which are produced on a large scale complying with good manufacturing practices (GMP).

In 2004 Ghana implemented ASAQ as national first-line treatment for uncomplicated *P. falciparum *malaria, after experts had reviewed data on efficacy of chloroquine (CQ) indicating widespread CQ resistance throughout the country [6]. At that time, limited data on the efficacy and safety of ASAQ and AL were available from sub-Saharan Africa [7,8]. Subsequently, overdosing of locally produced prepacked ASAQ for use in adults resulted in severe toxicity in several cases and fuelled the call for alternative drugs with better tolerability, e.g. assumed for AL. Clinical trials in Uganda showed high efficacy and good tolerability of AL when used as a six-dose therapy for uncomplicated malaria in children, with a risk of treatment failure due to recrudescence of less than 2% [9,10]. The high cure rates of AL were confirmed in studies from Tanzania [11-13], suggesting that this combination is still effective in areas with considerable resistance to other antimalarial drugs.

However, despite the promising results of AL, there are substantial limitations of this regimen, including the twice-daily dosing schedule, the preferable administration with fatty food, and selection for drug-resistant parasites. Although adequate clinical and parasitological responses in Ugandan children following unsupervised treatment were similar to supervised treatment, other studies showed that unsupervised therapy with AL resulted in lower plasma levels of lumefantrine with an increased risk for early reinfection [9,10].

The aim of this study was to compare the effectiveness, tolerability and acceptance of unsupervised AL to that of unsupervised ASAQ in an area of intense malaria transmission in Ghana.

## Methods

### Study sites and participants

This randomized, open-label trial was conducted between October, 2006 and September, 2007 at two district hospitals within the Ashanti region of Ghana, the Child Welfare Clinic of the Agogo Presbyterian Hospital, Asante Akim North district, and the outpatient department of Agona Government Hospital, Afigya Sekyere district. At both sites, the predominant ethnic group is Ashanti, minorities are Fanti, Ewe, Ga and Dagomba.

The majority of the population subsists on small-scale crop farming, notably trading in general goods also plays an important role. The vegetation in the study area is partly tropical woodland, partly savanna. The area is classified as holoendemic and malaria transmission occurs perennial with seasonal peaks [14]. The predominant species is *P. falciparum *and the principal vectors are mosquitoes of the *Anopheles gambiae *complex and *A. funestus*. According to annual reports of the District Health Directorate more than 20% of hospital consultations are attributed to malaria, and malaria-related mortality is reported to be around 25% in children less than five years of age. Ownership of insecticide-treated bednets (ITNs) in the region was reported to be only 1.6%, but subsidizing and education programmes are ongoing [15].

### Study procedures

Children aged 6–59 months with measured axillary body temperature ≥ 37.5°C and/or history of fever within the previous 24 hours presenting at the hospitals were initially seen by the study physician. After obtaining capillary blood samples, thick and thin blood films were prepared, and Diff-Quik^® ^(Dade-Behring, Switzerland) stained thick films were microscopically examined for the presence of malaria parasites. Parents/guardians of slide-positive children with *P. falciparum *monoinfection, a parasite density between 2,000 and 200,000 asexual parasites/μl, and without signs of severe malnutrition (weight-for-age < -3 SD of the median of the WHO child growth standard) or any other severe underlying disease were asked to participate in the study if they were permanent residents in the area.

Further inclusion criteria were 1) absence of general danger signs or signs of severe malaria according to WHO [16], 2) no intake of antibiotics and no adequate antimalarial treatment within the previous 7 days, 3) absence of a history of hypersensitivity to any of the study drugs, 4) ability to tolerate oral therapy, and 5) informed consent provided by a parent/guardian. Before enrolment, the aims and procedures of the study were explained to the parent/guardian and his/her understanding was confirmed by interview before written or, in case of illiteracy, thumb-printed consent was obtained in the presence of an unbiased witness.

Children were randomly assigned to receive either ASAQ or AL. Before drug application a venous blood sample was taken to measure leukocyte counts as indicator for a bacterial co-infection. Treatment allocation was based on a computer-generated list with randomization of individuals in blocks of ten.

Co-blister packed ASAQ [Arsucam^® ^(50 mg AS/white tablet and 153 mg AQ/yellow tablet), Sanofi-Aventis, Paris, France] were given weight-adjusted [5 – <10 kg (AS 1/2 tablet + AQ 1/2 tablet), 10 – <21 kg (AS 1 tablet + AQ 1 tablet), and 21 – <40 kg (AS 2 tablets + AQ 2 tablets] once daily for 3 days, as recommended by the manufacturer.

AL [Coartem^® ^(20 mg artemether/120 mg lumefantrine fixed-dose combination tablets), Novartis, Basel, Switzerland] were administered twice daily as three-day, six-dose regimen according to body weight [5 – <15 kg (1 tablet), 15 – <25 kg (2 tablets), or 25 – <35 kg (3 tablets)], the second dose 8 hours after the first dose, according to manufacturer guidelines.

After enrolment, evaluation and consenting, participating children received the first dose of the individually allocated treatment (in sealed and numbered, opaque envelopes) directly observed by a study nurse in a separate cabinet, while study physicians were blinded to treatment allocation. Prior to administration the tablets were crushed and mixed with water.

Treated children were observed for 30 minutes and the dose was readministered if vomiting occured. Children who repeatedly vomited their first dose of study medication were referred for alternative management. Subsequently, the treatment schedule was explained to the parents, who were strongly encouraged to resume normal diet and give the study medication with fat-containing food or during nursing if the participating child was breastfed. The three-day treatment course was then completed at home.

Children received paracetamol for treatment of febrile symptoms. Those with haemoglobin concentrations of less than 10 g/dl received ferrous sulfate, folic acid and anthelmintic treatment (if appropriate) according to national guidelines.

Participants were asked to attend regular follow-up visits on days 3, 7, 14, 28. Except for day 3, postponement of one day was tolerated. Parents/guardians were encouraged to come to the clinic whenever a child felt sick, and to return immediately to the hospital in case danger signs and/or deterioration of the clinical status were observed during treatment, or in case a treatment dose was vomited.

Study participants were visited actively at home by members of the study team and taken to the clinic if absent on a scheduled visit day. On each visit a standardized medical history and medical examination Case Report Form was completed. Capillary blood was obtained by finger-prick for thick and thin blood films, a drop of blood was stored on filter paper (Whatman^®^, Kent, UK) and a photometric measurement of haemoglobin (HemoCue^® ^Ltd., Derbyshire, UK) was performed. Full blood counts of venous blood samples were done with an automated haematology analyzer on day 0 (Kx-21N, Sysmex^® ^Europe GmbH, Norderstedt, Germany). Blood smears were stained with Giemsa and parasite densities were determined by counting asexual parasites per 200 white blood cells (or 500, if the count was less than 10 parasites per 200 white blood cells), assuming a white blood cell count of 8,000/μl. Smears were considered negative if no asexual parasite was seen after review of at least 100 high-power fields. Parasite species were determined by examination of thin films. For quality control slides of >10% of the enrolled patients were re-examined by a second microscopist who was blinded for the patient number, day of follow-up, and former microscopic results. After comparing the new with the original outcome classifications, no discrepancies were found. Children who did not tolerate oral therapy (repeated vomiting after initial study drug application), with mixed plasmodial infections, hyperparasitaemia (>200,000 asexual parasites/μl), or leukocytosis (>15,000 leukocytes/μl) were subsequently excluded from the analysis.

### Primary outcomes

Treatment outcomes were defined according to WHO guidelines (2003) as early treatment failure (ETF): danger signs or complicated malaria or failure to adequately respond to therapy on days 0–3; late clinical failure (LCF): danger signs or complicated malaria or fever/history of fever in the previous 24 hours and parasitaemia on days 4–28 without previously meeting criteria for ETF; late parasitological failure (LPF): asymptomatic parasitaemia day 4–28 without previously meeting criteria for ETF or LCF; and adequate clinical and parasitological response (ACPR): absence of parasitaemia on day 28 without previously meeting criteria for ETF, LCF, or LPF [17]. All patients classified as treatment failures received rescue treatment with quinine (10 mg/kg three times daily for 7 days).

The primary effectiveness outcomes were the 28-day risks of recurrent symptomatic malaria (ETF and LCF) and recurrent parasitaemia (ETF, LCF and LPF), unadjusted and adjusted by genotyping.

### Parasite genotyping

Recrudescent infections were distinguished from reinfections by nested-PCR of the variable block 2 region of the merozoite surface proteine-1 gene (*msp-1*, belonging to the three allele families K1, MAD20 and RO33) and of the polymorphic block 3 region of the merozoite surface proteine-2 gene (*msp-2*, 3D7 and FC27 allelic families), after extracting DNA from filter papers of paired samples (samples collected on day 0 and the day of failure) following the manufacturer's boiling protocol. Subsequently, amplicon lengths were analysed by capillary electrophoresis using a DNA analyzer (ABI Prism^® ^3100 Genetic Analyzer, Applied Biosystems, CA, USA) with GeneMapper™ ID software (BioType, Dresden, Germany) as described elsewhere [14]. A recurrent parasitaemia was categorized as reinfection if none of the *msp1/msp2 *amplicons in polyclonal infections had identical lengths. Accordingly, recrudescence was defined as the presence of at least one *msp1/msp2 *amplicon of identical length.

### Secondary outcomes

Secondary outcome measures were the change in the mean haemoglobin concentration from day 0 to day 28, the comparative tolerability, and the acceptance of the treatment regimen. The comparative tolerability was assessed by the risk of occurrence of an adverse event (AE), classified by means of the Division of Microbiology and Infectious Diseases pediatric toxicity tables [18] as mild (grade 1), moderate (grade 2), severe (grade 3), or serious (grade 4, SAE). A serious adverse event was defined as any AE resulting in death or in persistent or significant disability/incapacity, being life-threatening, requiring hospitalization or significant medical intervention to prevent serious outcome. For each AE, causality was assessed as recommended by the WHO [19].

Acceptance levels were assessed by asking the parents/guardians three standardized questions about the study treatment (after completion of the treatment course), i.e. a) how they appraised the treatment schedule, b) how they liked the packaging of the drug, and c) how they found the administration of the drug. Answers were scored with 1 point (difficult/bad), 2 (fairly difficult/fair), 3 (easy/good), or 4 (very easy/very good) points, and were compared using rank sum tests.

### Sample size

The sample size was calculated to get at least 90% power to detect a 10% difference of the risk of treatment failure (defined as the number of patients with clinical cure and clearance of asexual *P. falciparum *parasitaemia without subsequent PCR-confirmed recrudescence by day 28) between the two study arms at a 5% significance level. A cure rate of more than 95% in one of the two treatment arms was anticipated. Expecting a dropout rate of not more than 10%, the targeted number of participants in each study arm was 228.

### Data analysis

Data generated during the trial were recorded in the Case Report Form (CRF) of the respective patient and timely double-entered into two separate databases (4^th ^Dimension^®^, 2004 version 8.0.4, 4D, Neufahrn, Germany) with variable ranges and internal consistency controls before files were locked. Final analyses were done with Stata/IC™ version 10.0 (StataCorp, College Station, TX, USA).

Per-protocol analysis included all patients who matched all inclusion criteria, were properly randomized, had received the study drugs according to the protocol, and for whom data were available on the study end-point without protocol violation during the follow-up time. Intention-to-treat analysis comprised all randomized patients fulfilling the inclusion criteria without repeated vomiting after the first study drug administration. Characteristics of both study arms were compared by contingency (χ^2^) and nonparametric (Wilcoxon) tests, and for evaluation of the acceptance questionnaires Wilcoxon rank-sum tests were performed with scores allocated to the potential answers. Cox regression was used to calculate ACPR, LCF and occurrence of adverse events. P values < 0.05 were considered significant.

### Ethical considerations

All clinical investigations were conducted according to the principles of the Helsinki Declaration. The protocol was approved by the Committee on Human Research Publications and Ethics of the School of Medical Sciences of the Kwame Nkrumah University of Science and Technology, Kumasi, Ghana, and the Ethics Committee of the Medical Association Hamburg, Germany. In addition to the immediate notification of SAEs, all AEs were reported on a monthly basis to the data and safety monitoring board (DSMB). An experienced clinical trial monitor audited the trial that was registered at  (NCT00374205).

## Results

### Baseline data and participant flow

**Figure 1:**
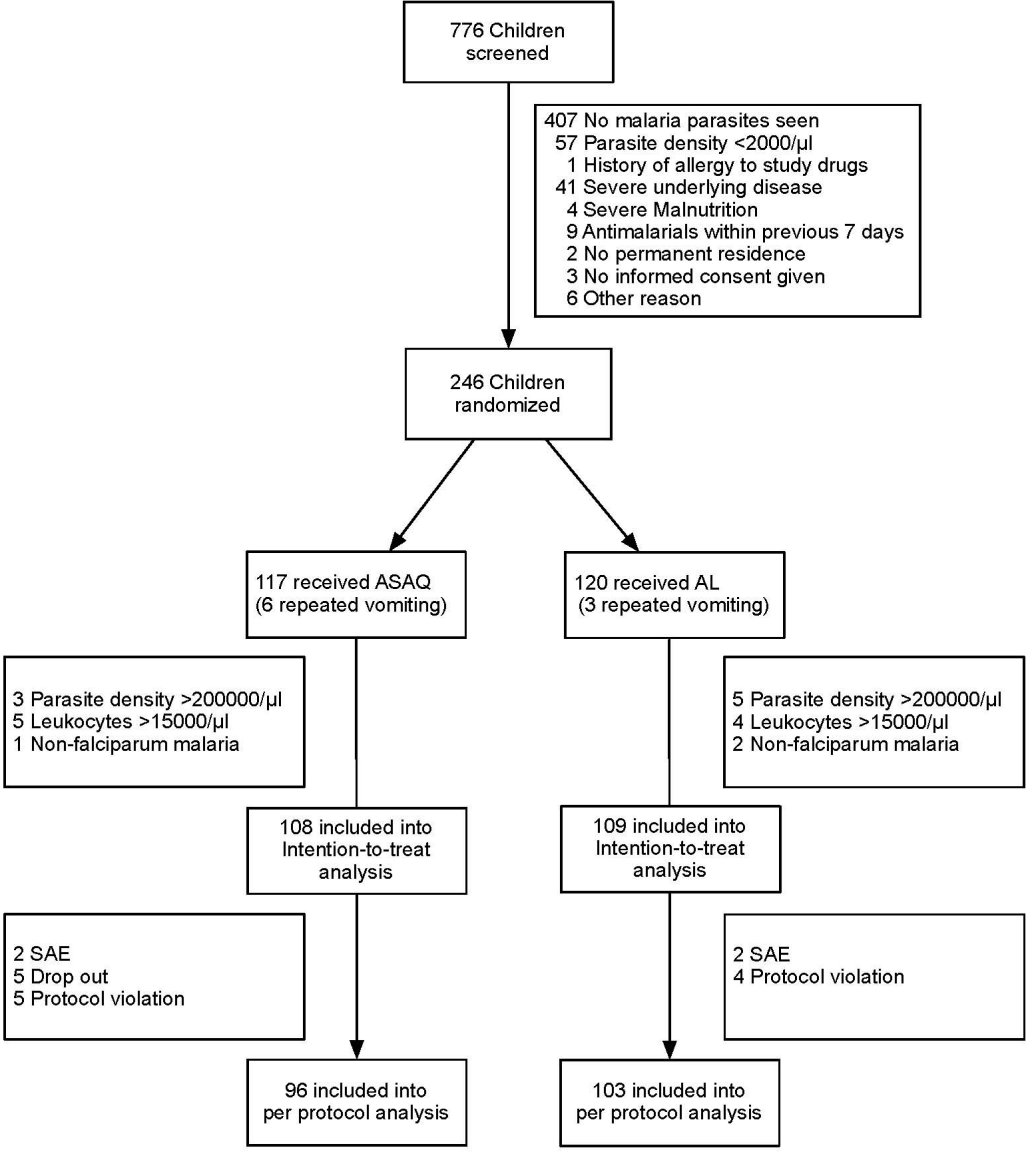
**Trial profile**.

The study started in October 2006 and was stopped in September 2007. At that time, a total of 776 children were screened and 246 were randomized (Figure 1). Adjusted and unadjusted treatment outcome was available for 217 children in the intention-to-treat (ITT) and for 199 children in the per-protocol (PP) analysis. There was no significant difference in baseline characteristics of the two treatment groups (Table 1). After initial randomization 9 children were excluded due to repeated vomiting of the study drugs (they subsequently received alternative therapy). According to the study protocol, another 20 individuals had to be excluded from analysis because of the detection of >200,000 asexual parasites/μl in the Giemsa-stained thick film, or of non-falciparum parasites, or of leukocyte counts >15,000/μl (Figure 1).

**Table 1:** Baseline characteristics of randomized study participants

**Characteristic**	**ASAQ**	**AL**	**p value**^a^
	n = 123	n = 123	
Patients, n (percentage)			
Agogo site	41 (33.3%)	39 (31.7%)	0.785
Agona site	82 (66.7%)	84 (68.3%)	

Sex (male: female), n	68: 55	65: 58	0.701
Age, months (SD)	32.1 (± 16.1)	30.7 (± 13.7)	0.458
Weight-for-age^b^, z-score (SD)	-0.93 (± 1.2)	-0.81 (± 1.1)	0.414
Axillary temperature,°C (SD)	38.1 (± 0.98)	38.0 (± 1.1)	0.891
Parasite density^c^,/μl	38864	34255	0.572
Haemoglobin, g/dl (SD)	9.9 (± 1.9)	9.6 (± 1.7)	0.133

**Please note**:

Data are mean values (SD), unless otherwise indicated.

^a ^Determined using Wilcoxon test or χ^2 ^test. None of the parameters in both study arms was significantly different.

^b ^According to the WHO child growth standards.

^c ^Geometric mean; parasite densities were determined from Giemsa-stained thick blood films.

ASAQ, artesunate plus amodiaquine; AL, artemether-lumefantrine

SD, standard deviation

### Primary effectiveness outcome

**Figure 2:**
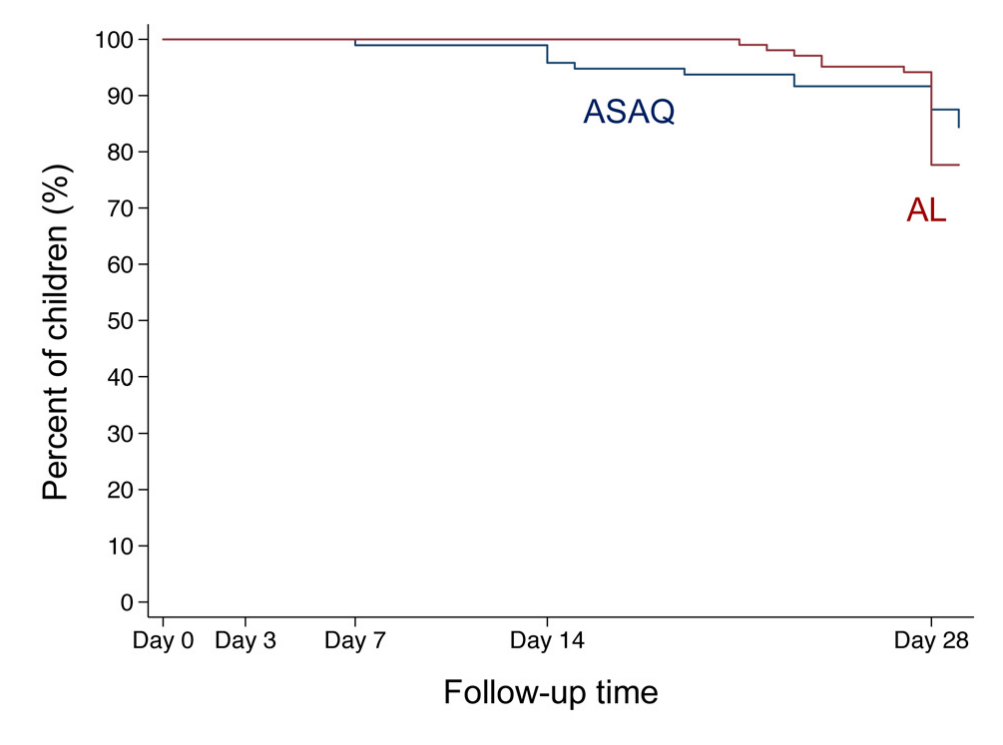
**Kaplan-Meier survival curve of adequate clinical and parasitological response (ACPR)**. The figure shows the percentage of children with ACPR during the follow-up time of 28 days (in some plus 1 day) separated by treatment group according to the per-protocol analysis. The red line indicates children treated with artemether-lumefantrine (AL), the blue line indicates children treated with artesunate plus amodiaquine (ASAQ).

**Figure 3:**
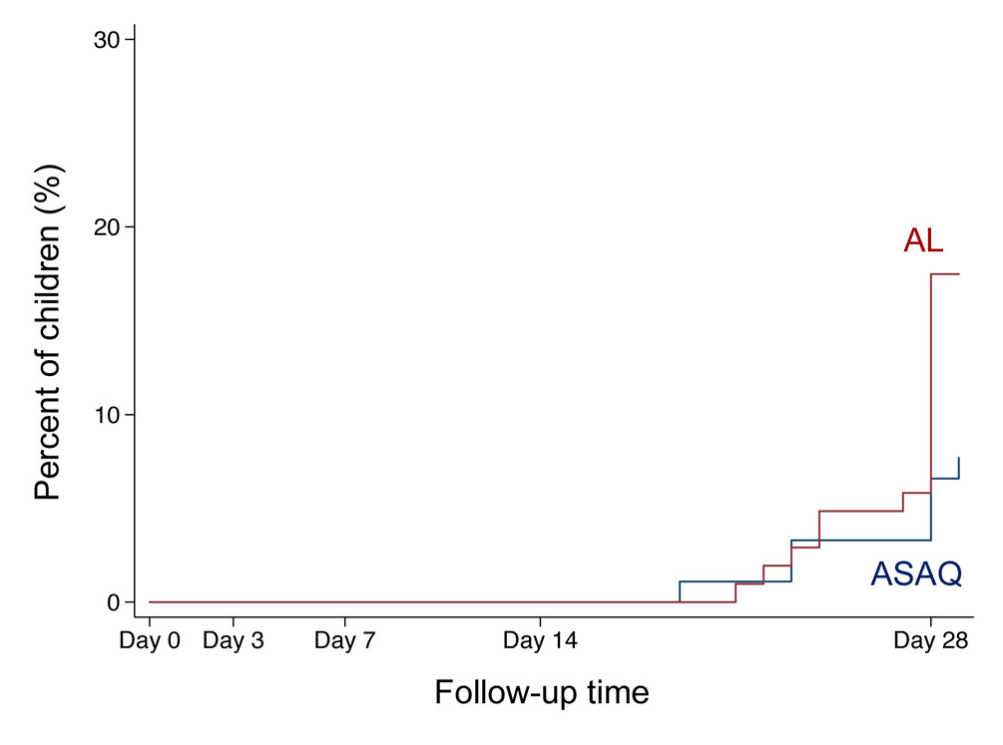
**Kaplan-Meier failure curve of late clinical failures (LCF)**. The figure shows the percentage of children with LCF during the follow-up time of 28 days (in some plus 1 day) separated by treatment group according to the per-protocol analysis. The red line indicates children treated with artemether-lumefantrine (AL), the blue line indicates children treated with artesunate plus amodiaquine (ASAQ).

No ETF was observed. Adequate clinical and parasitological responses were similarly high in both treatment groups (Table 2). The per-protocol Kaplan-Meier survival curves are displayed in Figure 2. However, LCF rates by day 28 were significantly higher in AL-treated children than in ASAQ-treated children in both, the per-protocol analysis (17.5% and 7.3%, respectively; Hazard Ratio 2.41, 95% CI 1.00–5.79, p < 0.05; Figure 3) and in the intention-to-treat analysis (16.5% and 6.5%, respectively; HR 2.44, 95% CI 1.02–5.85, p < 0.05). A trend towards higher LCF rates was also seen after exclusion of reinfections (Table 2).

**Table 2:** Primary treatment outcomes

	**Treatment**			
		
**Outcome**	**ASAQ**	**AL**	**Hazard ratio** **(95% CI)**	**p value**^a^
**Per protocol**^b^	**n = 96**	**n = 103**		
Recurrent parasitaemia	15 (15.6%)	23 (22.3%)	1.49 (0.77–2.87)	0.233
Recurrent parasitaemia (PCR corrected)	7 (7.3%)	12 (11.7%)	1.71 (0.67–4.38)	0.265
ETF	0	0		
LCF	7 (7.3%)	18 (17.5%)	2.41 (1.003–5.79)	0.049
LCF (PCR corrected)	3 (3.1%)	10 (9.7%)	3.25 (0.89–11.92)	0.075
LPF	8 (8.3%)	5 (4.9%)	0.64 (0.21–1.99)	0.443
LPF (PCR corrected)	4 (4.2%)	2 (1.9%)	0.52 (0.09–2.87)	0.452
ACPR	81 (84.4%)	80 (77.7%)	0.96 (0.70–1.31)	0.792
ACPR (PCR corrected)	88 (91.7%)	91 (88.3%)	1 (0.74–1.34)	0.986

**Intention to treat**^c^	**n = 108**	**n = 109**		
Recurrent parasitaemia	15 (13.9%)	23 (21.1%)	1.51 (0.78–2.90)	0.218
Recurrent parasitaemia (PCR corrected)	7 (6.5%)	12 (11.0%)	1.72 (0.67–4.41)	0.256
ETF	0	0		
LCF	7 (6.5%)	18 (16.5%)	2.44 (1.02–5.85)	0.046
LCF (PCR corrected)	3 (2.8%)	10 (9.2%)	3.27 (0.89–11.96)	0.073
LPF	8 (7.4%)	5 (4.6%)	0.66 (0.21–2.04)	0.409
LPF (PCR corrected)	4 (3.7%)	2 (1.8%)	0.53 (0.10–2.94)	0.468
ACPR	88 (81.5%)	86 (78.9%)	0.96 (0.71–1.30)	0.813
ACPR (PCR corrected)	95 (88.0%)	97 (89.0%)	1.00 (0.75–1.33)	0.988

**Please note**:

^a ^Tested by Cox regression.

^b ^Included were all patients who matched the inclusion criteria without violating the protocol.

^c ^Included were all patients who matched all inclusion criteria without repeated vomiting after the first study drug administration.

CI, convidence interval; ASAQ, artesunate plus  amodiaquine;  AL, artemether-lumefantrine; ETF, early treatment failure;  LCF, late clinical failure; LPF, late parasitological failure; ACPR,  adequate clinical and parasitological response

### Secondary effectiveness outcome

#### Haematologic recovery

Until September 2007, more than 50% of the study participants developed anemia with haemoglobin values that were classified as AEs after day 0. Therefore, in agreement with the study protocol, the DSMB recommended the discontinuation of the trial. The final analysis showed that haemoglobin levels on day 0 were not different in both treatment arms, and haematologic recovery by day 28 was adequate and similar after AL and after ASAQ treatment [mean increase of haemoglobin AL group: 1.17 g/dl (95% CI 0.82–1.53); ASAQ group: 1.38 g/dl (95% CI 1.00–1.75); p = 0.44].

#### Tolerability

Both study drugs were well tolerated, and no drug-related SAE occurred during the study. There were four SAEs (two in each treatment arm, all were classified as "unlikely related to treatment"), i.e. one was a mild acute asthmatic attack on day 28, one a febrile convulsion on day 1, one child received concomitant treatment for suspected enteritic bacterial coinfection on day 3 and one child was extensively monitored due to a measured haemoglobin level of 4.8 g/dl on day 3 but subsequently needed no blood transfusion. Adverse events of grade 1–3 were, regarding their nature, severity, and relation to the study drug, similarly distributed in the study groups (Table 3).

**Table 3:** Adverse events

**Type of adverse event**	**ASAQ**	**AL**		
	**n = 123**	**n = 123**	**OR**	**p value**^a^
Vomiting of study drugs:				
at least once	15	11	0.71 (0.31–1.61)	0.408
twice	6	3	0.49 (0.12–1.99)	0.318

	**n = 117**	**n = 120**	**RR**	**p value**^b^
Deaths/hospitalizations	0	0		
Anaemia^c^	82	78	0.90 (0.66–1.23)	0.511
Respiratory symptoms	27	25	0.90 (0.52–1.54)	0.692
Gastrointestinal symptoms	12	10	0.78 (0.34–1.81)	0.565
Dermatological symptoms	3	2	0.63 (0.10–3.76)	0.610
Other symptoms	2	2	0.95 (0.13–6.72)	0.956

**Severity of adverse event**				

	**n = 117**	**n = 120**	**RR**	**p value**^b^
Mild	57	46	0.72 (0.49–1.07)	0.104
Moderate	45	54	1.19 (0.80–1.77)	0.382
Severe	15	22	1.45 (0.75–2.79)	0.268
SAE	2	2	0.96 (0.13–6.80)	0.966

**Please note**:

Adverse events were classified by means of the Division of Microbiology and Infectious Diseases pediatric toxicity tables [18].

^a ^Tested by logistic regression.

^b ^Tested by Cox regression.

^c ^Defined as Hb concentration <10 g/dl (age <2 years) and <11 g/dl (age >2 years).

ASAQ, artesunate plus amodiaquine; AL, artemether-lumefantrine; OR, odds ratio; RR, relative risk

SAE, serious adverse even

#### Acceptance

More parents/guardians in the ASAQ group described the treatment schedule as being very easy to apply, as compared to those of the AL group (77.4% and 55.5%, respectively; Wilcoxon test, p < 0.0005). This resulted in a slightly higher overall rank-score for ASAQ, compared to AL (10.6 and 10.3, respectively; p < 0.05), although packaging and administration of the study drugs were appraised similarly positive in both groups (see additional file 1 for the original questions and data used to perform this analysis).

On inquiry, almost all parents/guardians quoted that they would follow the same type of treatment again or advise a friend or family member to use the respective drug.

## Discussion

In this randomized effectiveness trial from Ghana cure rates of ASAQ and AL for the treatment of uncomplicated falciparum malaria were around 90%, qualifying both ACTs as highly effective in children. LCFs until day 28 uncorrected and, as a trend, corrected for reinfections were more frequently observed in AL-treated children than in ASAQ-treated individuals. This observation of similar ACPRs but higher LCFs after treatment with AL, could reflect a difference in the prevention of recurrent clinical malaria episodes between the two ACTs.

One limitation of the study is its premature termination due to the high frequency of anemia cases (>50%). The discontinuation was necessary through the stopping rules of the study protocol despite the fact that the occurrence of anemia was not different in both study arms and was not related to treatment. Accordingly, the analysis was performed with a decreased number of study participants providing a power of 65% to detect differences between the study arms. Therefore, the lack of a significantly different effectiveness in one of the study arms has to be interpreted with caution. Nevertheless, cure rates could be provided within the given confidence intervals and the findings of different LCFs in both arms are valid independent of the estimated power.

Another limitation of the study is the restriction of the follow-up period to 28 days bearing the risk of underestimation of treatment failures. Although logistically difficult, only longitudinal clinical trials with follow-up time over several months are appropriate for comparing the benefits and risks of different antimalarial drugs, especially in highly endemic areas [20]. Recently, an efficacy trial comparing ASAQ and AL was performed in Ghana with a follow-up time of one whole year showing high day-28 ACPRs of 95.3% and 94.2%, respectively, with no difference in the occurrence of reinfections or adverse events [21]. However, the differentiation between reinfection and resistance is even more difficult with increasing follow-up periods.

With respect to the interpretation of PCR-controlled cure rates, it has to be considered, that the definition of reinfections is critical for the assessment of drug efficacy and effectiveness. In the study presented here, a recrudescence was defined as a recurrent *P. falciparum *parasitaemia with detection of at least one *msp1/msp2 *amplicon of identical length within the 28-day follow-up period compared to amplicons found on day 0. This definition was chosen very stringent to identify all possible treatment failures. Remarkably, although most infections in our study area are multiclonal and the genetic diversity is high [14], some amplicons occur at high frequency. In principle, this increases the likelihood that amplicons with identical lengths were found in reinfections, leading to an overestimation of recrudescences and, consequently, treatment failures. Recently, the pitfalls of different genotyping approaches in antimalarial drug trials were analysed in relation to the transmission intensity [22]. The authors concluded that, especially regarding high endemicity settings, methods to overcome the inaccuracy of genotyping to distinguish recrudescenses from new infections were limited and complex, and comparative results should be interpreted, rather to rely on the absolute efficacy of a respective drug. But not only treatment efficacy, also post-treatment prophylaxis is of major concern in areas of intense malaria transmission, where timely recurrent symptomatic malaria episodes require re-treatment, and subsequently influence the acquisition of immunity and increase the risk of development of drug resistance [23].

In accordance with these assumptions and in addition to the study mentioned above a recent comparative efficacy study in Ghanaian children showed lower parasitological and clinical failure rates in the ASAQ compared to the AL arm [24]. Another trial from Ghana showed a high PCR-corrected efficacy of ASAQ and AL of 100% and 97.5%, respectively [25]. Together with recently published efficacy data the presented study shows that ASAQ is highly efficacious, if not superior to AL, in treating uncomplicated malaria in Ghana.

These results are in contrast to most comparative efficacy trials from Central and East Africa. In the Republic of Congo, in Uganda and Tanzania including Zanzibar, AL showed higher efficacy than ASAQ, especially a higher potency in preventing reinfections [11-13,26-28]. In one study from an area with holoendemic malaria transmission in Uganda, both regimens, ASAQ and AL, were highly efficacious for clearance of infections but the risk of reinfection by day 42 was found to be high in both study arms. In that study, although initial cure rates were above 90%, 66% of ASAQ-treated and only 50% of AL-treated patients developed recurrent parasitaemia within 28 days [28]. Only in Nigeria, one of a few West-African countries from where data is available, the efficacies of ASAQ and AL after supervised treatment were similarly high, with PCR-corrected cure rates of 98.4% and 100%, respectively [29], while uncorrected ACPRs were 87.0% and 82.5%, respectively [30].

To explain the observed differences, one has to consider that not only the prevalences of molecular markers for ASAQ and AL drug resistance vary in different geographical regions [31,32], but also other parasite characteristics, the genetic background of the host and multiple environmental factors.

The lower effectiveness of AL in our trial compared to previous studies might be explained by unreliable or incorrect drug intake when unobserved. Furthermore, intestinal absorption of lumefantrine, the long-acting partner drug in AL, can be critically influenced by concomitant intake of fat-containing food [33]. Nevertheless, the only other comparative effectiveness study performed in Tanzania showed superiority of AL over ASAQ [11]. In the present study, a fat-containing diet was emphatically advised throughout, yet it forms a possible source of treatment failure due to sub-therapeutic plasma drug concentrations. AL tablets need to be taken twice daily, with equal number of tablets per dose. Therefore, skipping single doses, not taking them entirely, or not coadministering fat-containing food could also have resulted in a higher frequency of subtherapeutic drug levels in the AL arm. Nevertheless, good compliance with AL treatment seems to be possible, as shown in a trial on home management of malaria in Ghana [34]. In Gabon, the outcome after once-daily ASAQ treatment was significantly improved by direct observation [35]. In this context, it seems reasonable to hypothesize that the fixed-dose combination of artesunate plus amodiaquine, which is currently replacing the co-blister packed ASAQ, will further increase therapeutic adherence and treatment effectiveness. Indeed, the acceptance of both ACTs was high and only few caregivers reported general problems with the study medications.

The trial was discontinued due to a high frequency of anemia cases in our study population. Nevertheless, anemia occurred equally often in both study arms and haematologic recovery was similar and adequate in both arms. The safety profiles of the study drugs were good and our findings are in agreement with previous drug safety monitorings in Senegal [36,37]. It is noteworthy that six ASAQ-treated children and only three children in the AL arm had to be excluded after repeated vomiting of the study drug. Though not significant, this could be an indicator of a poorer tolerability of ASAQ, which has also been shown in a recently published trial from Ghana [24]. Pharmacokinetics after combined administration of artesunate and amodiaquine, unlike the extensive investigations of AL, were just recently systematically tested in fifteen healthy volunteers [38]. Drug interactions, frequency and nature of adverse events, namely severe transaminitis (n = 1), neutropenia (n = 2), and hypersensitivity (n = 1), were called a matter of concern. The risk of ASAQ-induced neutropenia was reported to be increased in HIV-positive individuals, especially when concomitantly treated with antiretrovirals [39]. Therefore, despite its high efficacy, ASAQ for combination therapy has to be carefully monitored in areas with high HIV prevalence.

## Conclusion

ASAQ and AL were both well tolerated and highly effective for treatment of Ghanaian children with uncomplicated falciparum malaria. Together with recent efficacy data, the more effective prevention of LCFs after unobserved ASAQ compared to AL treatment, affirms its appropriate role as national first-line therapy in Ghana. Monitoring of efficacy, safety, and drug resistance markers are essential to guide national treatment policies, however, further comparative effectiveness studies of ACTs are needed in the West African region.

## Abbreviations

ACPR: adequate clinical and parasitological response; ACT: artemisinin-based combination therapy; AE: adverse event; AL: artemether-lumefantrine; AQ: amodiaquine; AS: artesunate; ASAQ: artesunate plus amodiaquine;°C: degree Centigrade; CI: confidence interval; CQ: chloroquine; dl: deciliter; DNA: desoxyribonucleic acid; DSMB: data and safety monitoring board; ETF: early treatment failure; g: gram; Hb: haemoglobin; HIV: human immunodeficiency virus; HR: hazard ratio; ITT: intention-to-treat; LCF: late clinical failure; LPF: late parasitological failure; mg: milligram; μl: microliter; *msp*: merozoite surface protein; n: number; p: probability; PCR: polymerase chain reaction; *P. f.*: *Plasmodium falciparum; *PP: per-protocol; SAE: serious adverse event; SD: standard deviation; WHO: World Health Organization.

## Competing interests

The authors declare that they have no competing interests.

## Authors' contributions

All authors participated in design, implementation, analysis or interpretation of the study. RK designed the study. RK, PK and JM were involved in all phases of the study and have full access to all the data in the study. RK takes responsibility for the integrity of the data and the accuracy of the data analysis. DA was the Principal Investigator and supervised the study. PK, MB, SAD, WNT and SAM were responsible for conduction of field studies and coordination of study procedures. Further acquisition and analysis of data was performed by WL, JV, MN, BK and BH. Analysis of data was led by RK, WL and JM. The manuscript was drafted by RK and PK and substantial input came from all investigators. All authors read and approved the final manuscript.

## Supplementary Material

Additional file 1 Questions asked after completion of the 3-day treatment course to evaluate treatment acceptance of ASAQ and AL. Answers of parents/guardians were scored with 1 point (difficult/bad), 2 (fairly difficult/fair), 3 (easy/good), or 4 (very easy/very good) points, and were compared using Wilcoxon rank sum tests. Click here for file

## References

[B1] White NJ, Nosten F, Looareesuwan S, Watkins WM, Marsh K, Snow RW, Kokwaro G, Ouma J, Hien TT, Molyneux ME, Taylor TE, Newbold CI, Ruebush TK, Danis M, Greenwood BM, Anderson RM, Olliaro P (1999). Averting a malaria disaster. Lancet.

[B2] Kremsner P, Krishna S (2004). Antimalarial Combinations. Lancet.

[B3] Nosten F, van Vugt M, Price R, Luxemburger C, Thway KL, Brockman A, McGready R, ter Kuile F, Looareesuwan S, White NJ (2000). Effects of artesunate-mefloquine combination on incidence of *Plasmodium falciparum *malaria and mefloquine resistance in western Thailand: a prospective study. Lancet.

[B4] Breman JG (2001). The ears of the hippopotamus: manifestations, determinants, and estimates of the malaria burden. Am J Trop Med Hyg.

[B5] World Health Organization (2001). Antimalarial drug combination therapy: report of a WHO Technical Consultation Geneva.

[B6] World Health Organization The new anti malaria drug policy for Ghana. http://www.who.int/countries/gha/news/2006/anti.malaria.drug.policy/en/.

[B7] Adjuik M, Agnamey P, Babiker A, Borrmann S, Brasseur P, Cisse M, Cobelens F, Diallo S, Faucher JF, Garner P, Gikunda S, Kremsner PG, Krishna S, Lell B, Loolpapit M, Matsiegui PB, Missinou MA, Mwanza J, Ntoumi F, Olliaro P, Osimbo P, Rezbach P, Some E, Taylor WR (2002). Amodiaquine-artesunate versus amodiaquine for uncomplicated *Plasmodium falciparum *malaria in African children: a randomised multicentre trial. Lancet.

[B8] Omari AA, Preston C, Garner P (2003). Artemether-lumefantrine for treating uncomplicated falciparum malaria. Cochrane Database Syst Rev.

[B9] Piola P, Fogg C, Bajunirwe F, Biraro S, Grandesso F, Ruzagira E, Babigumira J, Kigozi I, Kiguli J, Kyomuhendo J, Ferradini L, Taylor W, Checchi F, Guthmann JP (2005). Supervised versus unsupervised intake of six-dose artemether-lumefantrine for treatment of acute, uncomplicated *Plasmodium falciparum *malaria in Mbarara, Uganda: A randomised trial. Lancet.

[B10] Babigumira J, Kigozi I, Kiguli J, Kyomuhendo J, Ferradini LJ, Taylor WR, Guthmann JP (2006). Supervised versus unsupervised antimalarial treatment with six-dose artemether-lumefantrine: pharmacokinetic and dosage-related findings from a clinical trial in Uganda. Malar J.

[B11] Mutabingwa TK, Anthony D, Heller A, Hallett R, Ahmed J, Drakeley C, Greenwood BM, Whitty CJ (2005). Amodiaquine alone, amodiaquine+sulfadoxine-pyrimethamine, amodiaquine+artesunate, and artemether-lumefantrine for outpatient treatment of malaria in Tanzanian children: a four-arm randomised effectiveness trial. Lancet.

[B12] Mårtensson A, Strömberg J, Sisowath C, Msellem MI, Gil JP, Montgomery SM, Olliaro P, Ali AS, Björkman A (2005). Efficacy of artesunate plus amodiaquine versus that of artemether-lumefantrine for the treatment of uncomplicated childhood *Plasmodium falciparum *malaria in Zanzibar, Tanzania. Clin Infect Dis.

[B13] Kabanywanyi AM, Mwita A, Sumari D, Mandike R, Mugittu K, Abdulla S (2007). Efficacy and safety of artemisinin-based antimalarial in the treatment of uncomplicated malaria in children in southern Tanzania. Malar J.

[B14] Kobbe R, Neuhoff R, Marks F, Adjei S, Langefeld I, Adjei O, Meyer CG, May J (2006). Seasonal variation and high multiplicity of first *Plasmodium falciparum *infections in children from a holoendemic area in Ghana, West Africa. Trop Med Int Health.

[B15] Ghana Statistical Service (GSS), Noguchi Memorial Institute for Medical Research (NMIMR), and ORC Macro (2004). Ghana Demographic and Health Survey 2003 Calverton, Maryland.

[B16] World Health Organization (2000). Severe falciparum malaria. Trans R Soc Trop Med Hyg.

[B17] World Health Organization Assessment and monitoring of antimalarial drug efficacy for the treatment of uncomplicated falciparum malaria.

[B18] National Institutes of Health, National Institute of Allergy and Infectious Diseases Division of Microbiology and Infectious Diseases Pediatric Toxicity Tables – Draft, November 2007. http://www3.niaid.nih.gov/research/resources/DMIDClinRsrch/toxtables.htm.

[B19] Edwards IR, Biriell C (1994). Harmonisation in pharmacovigilance. Drug Safety.

[B20] Laufer MK, Djimdé AA, Plowe CV (2007). Monitoring and deterring drug-resistant malaria in the era of combination therapy. Am J Trop Med Hyg.

[B21] Badoe EV, Lamptey R, Goka BQ (2008). Amodiaquine-artesunate vs artemether-lumefantrine for uncomplicated malaria in Ghanaian children: a randomized efficacy and safety trial with one year follow-up. Malar J.

[B22] Greenhouse B, Dokomajilar C, Hubbard A, Rosenthal PJ, Dorsey G (2007). Impact of transmission intensity on the accuracy of genotyping to distinguish recrudescence from new infection in antimalarial clinical trials. Antimicrob Agents Chemother.

[B23] White NJ (2008). How antimalarial drug resistance affects post-treatment prophylaxis. Malar J.

[B24] Owusu-Agyei S, Asante KP, Owusu R, Adjuik M, Amenga-Etego S, Dosoo DK, Gyapong J, Greenwood B, Chandramohan D (2008). An open label, randomised trial of artesunate+amodiaquine, artesunate+chlorproguanil-dapsone and artemether-lumefantrine for the treatment of uncomplicated malaria. PLoS ONE.

[B25] Koram KA, Abuaku B, Duah N, Quashie N (2005). Comparative efficacy of antimalarial drugs including ACTs in the treatment of uncomplicated malaria among children under 5 years in Ghana. Acta Trop.

[B26] Broek I van den, Kitz C, Al Attas S, Libama F, Balasegaram M, Guthmann JP (2006). Efficacy of three artemisinin combination therapies for the treatment of uncomplicated Plasmodium falciparum malaria in the Republic of Congo. Malar J.

[B27] Bukirwa H, Yeka A, Kamya MR, Talisuna A, Banek K, Bakyaita N, Rwakimari JB, Rosenthal PJ, Wabwire-Mangen F, Dorsey G, Staedke SG (2006). Artemisinin combination therapies for treatment of uncomplicated malaria in Uganda. PLoS Clin Trials.

[B28] Dorsey G, Staedke S, Clark TD, Njama-Meya D, Nzarubara B, Maiteki-Sebuguzi C, Dokomajilar C, Kamya MR, Rosenthal PJ (2007). Combination therapy for uncomplicated falciparum malaria in Ugandan children: a randomized trial. JAMA.

[B29] Falade CO, Ogundele AO, Yusuf BO, Ademowo OG, Ladipo SM (2008). High efficacy of two artemisinin-based combinations (artemether-lumefantrine and artesunate plus amodiaquine) for acute uncomplicated malaria in Ibadan, Nigeria. Trop Med Int Health.

[B30] Meremikwu M, Alaribe A, Ejemot R, Oyo-Ita A, Ekenjoku J, Nwachukwu C, Ordu D, Ezedinachi E (2006). Artemether-lumefantrine versus artesunate plus amodiaquine for treating uncomplicated childhood malaria in Nigeria: randomized controlled trial. Malar J.

[B31] Happi CT, Gbotosho GO, Folarin OA, Bolaji OM, Sowunmi A, Kyle DE, Milhous W, Wirth DF, Oduola AM (2006). Association between mutations in *Plasmodium falciparum *chloroquine resistance transporter and *P. falciparum *multidrug resistance 1 genes and in vivo amodiaquine resistance in *P. falciparum *malaria-infected children in Nigeria. Am J Trop Med Hyg.

[B32] Sisowath C, Strömberg J, Mårtensson A, Msellem M, Obondo C, Björkman A, Gil JP (2005). In vivo selection of *Plasmodium falciparum *pfmdr1 86N coding alleles by artemether-lumefantrine (Coartem). J Infect Dis.

[B33] Ashley EA, Stepniewska K, Lindegårdh N, Annerberg A, Kham A, Brockman A, Singhasivanon P, White NJ, Nosten F (2007). How much fat is necessary to optimize lumefantrine oral bioavailability?. Trop Med Int Health.

[B34] Chinbuah AM, Gyapong JO, Pagnoni F, Wellington EK, Gyapong M (2006). Feasibility and acceptability of the use of artemether-lumefantrine in the home management of uncomplicated malaria in children 6–59 months old in Ghana. Trop Med Int Health.

[B35] Oyakhirome S, Pötschke M, Schwarz NG, Dörnemann J, Laengin M, Salazar CO, Lell B, Kun JF, Kremsner PG, Grobusch MP (2007). Artesunate-amodiaquine combination therapy for falciparum malaria in young Gabonese children. Malar J.

[B36] Faye B, Ndiaye JL, Ndiaye D, Dieng Y, Faye O, Gaye O (2007). Efficacy and tolerability of four antimalarial combinations in the treatment of uncomplicated *Plasmodium falciparum *malaria in Senegal. Malar J.

[B37] Brasseur P, Agnamey P, Gaye O, Vaillant M, Taylor WR, Olliaro PL (2007). Efficacy and safety of artesunate plus amodiaquine in routine use for the treatment of uncomplicated malaria in Casamance, southern Sénégal. Malar J.

[B38] Orrell C, Little F, Smith P, Folb P, Taylor W, Olliaro P, Barnes KI (2008). Pharmacokinetics and tolerability of artesunate and amodiaquine alone and in combination in healthy volunteers. Eur J Clin Pharmacol.

[B39] Gasasira AF, Kamya MR, Achan J, Mebrahtu T, Kalyango JN, Ruel T, Charlebois E, Staedke SG, Kekitiinwa A, Rosenthal PJ, Havlir D, Dorsey G (2008). High risk of neutropenia in HIV-infected children following treatment with artesunate plus amodiaquine for uncomplicated malaria in Uganda. Clin Inf Dis.

