# Supplementary material for: A randomized trial on effectiveness of artemether-lumefantrine versus artesunate plus amodiaquine for unsupervised treatment of uncomplicated Plasmodium falciparum malaria in Ghanaian children
Source: Malar J. 2008 Dec 19;7:261. doi: 10.1186/1475-2875-7-261 (PMC2625364; doi:10.1186/1475-2875-7-261)
Supplement: Additional file 1 — Questions asked after completion of the 3-day treatment course to evaluate treatment acceptance of ASAQ and AL. Answers of parents/guardians were scored with 1 point (difficult/bad), 2 (fairly difficult/fair), 3 (easy/good), or 4 (very easy/very good) points, and were compared using Wilcoxon rank sum tests. [file 1475-2875-7-261-S1.doc]

**Additional file 1. Evaluation of treatment acceptance**

|  | |  |  | **Treatment** | | | | | | | | | |  |
| --- | --- | --- | --- | --- | --- | --- | --- | --- | --- | --- | --- | --- | --- | --- |
|  |  |  |  | **ASAQ** | | | | | **AL** | | | | |  |
| **Question^a^** | | **Answers** |  | **n^c^** | **%** | **Mean** | **Rank sum^b^** | **Ex­pected** | **n^c^** | **%** | **Mean** | **Rank sum^b^** | **Ex­pected** | **p value** |
| **Q1** | |  |  | 115 | 100 | 3.8 | 15026 | 13512.5 | 119 | 100 | 3.5 | 12469 | 13982.5 | 0.0004 |
|  |  | Difficult = | 1 | 0 | 0 |  |  |  | 0 | 0 |  |  |  |  |
|  |  | Fairly difficult = | 2 | 0 | 0 |  |  |  | 1 | 0.8 |  |  |  |  |
|  |  | Easy = | 3 | 26 | 22.6 |  |  |  | 52 | 43.7 |  |  |  |  |
|  |  | Very easy = | 4 | 89 | 77.4 |  |  |  | 66 | 55.5 |  |  |  |  |
| **Q2** | |  |  | 115 | 100 | 3.5 | 13507.5 | 13512.5 | 119 | 100 | 3.5 | 13987.5 | 13982.5 | 0.9911 |
|  |  | Bad = | 1 | 0 | 0 |  |  |  | 0 | 0 |  |  |  |  |
|  |  | Fair = | 2 | 0 | 0 |  |  |  | 0 | 0 |  |  |  |  |
|  |  | Good = | 3 | 60 | 52.2 |  |  |  | 62 | 52.1 |  |  |  |  |
|  |  | Very good = | 4 | 55 | 47.8 |  |  |  | 57 | 47.9 |  |  |  |  |
| **Q3** | |  |  | 115 | 100 | 3.4 | 13782 | 13512.5 | 119 | 100 | 3.3 | 13713 | 13982.5 | 0.5641 |
|  |  | Difficult = | 1 | 0 | 0 |  |  |  | 0 | 0 |  |  |  |  |
|  |  | Fairly difficult = | 2 | 11 | 9.6 |  |  |  | 12 | 10.1 |  |  |  |  |
|  |  | Easy = | 3 | 52 | 45.2 |  |  |  | 58 | 48.7 |  |  |  |  |
|  |  | Very easy = | 4 | 52 | 45.2 |  |  |  | 49 | 41.2 |  |  |  |  |
|  |  |  |  |  |  |  |  |  |  |  |  |  |  |  |
| **Score** | |  |  | 115 | 100 | 10.6 | 14495 | 13512.5 | 119 | 100 | 10.3 | 13000 | 13982.5 | 0.0486 |
|  |  |  | 8 | 2 | 1.74 |  |  |  | 4 | 3.4 |  |  |  |  |
|  |  |  | 9 | 18 | 15.7 |  |  |  | 22 | 18.5 |  |  |  |  |
|  |  |  | 10 | 30 | 26.1 |  |  |  | 37 | 31.1 |  |  |  |  |
|  |  |  | 11 | 38 | 33.0 |  |  |  | 42 | 35.3 |  |  |  |  |
|  |  |  | 12 | 27 | 23.5 |  |  |  | 14 | 11.8 |  |  |  |  |

Q1: „How did you find the treatment schedule of the study drug?“, Q2: „How did you like the packaging of the drug?“, Q3: „How did you find the administration of the drug?“;

^a^ Remark: All questions were put in Twi, the predominant local language

^b^ Wilcoxon rank sum

^c^ Included were all participants for whom questionnaires were available.
